# Regional Anesthesia as Primary Strategy in Aesthetic Breast Augmentation Surgery: Clinical Outcomes in 627 Sedated Patients Undergoing Pectoral Nerves (PECS I–II) and Parasternal Blocks

**DOI:** 10.1007/s00266-026-05966-1

**Published:** 2026-06-03

**Authors:** Di Lorenzo Sara, Andrea Pio Cascino, Scalisi Alfio, Pulvirenti Giuseppe, Corradino Bartolo

**Affiliations:** 1 Plastic and Reconstructive Surgery, Department of Precision Medicine in Medical, Surgical and Critical Care (Me.Pre.C.C.), University of Palermo, Via del Vespro, 129, 90127 Palermo, Italy; 2 Well Surgery Clinic, Via Pietro Mascagni, 84, 95129 Catania, Italy

**Keywords:** Breast augmentation, Regional anesthesia, PECS block, Aesthetic surgery, Postoperative pain, Opioid-free

## Abstract

**Background:**

Post-operative pain after breast augmentation remains unpredictable, and regional anesthesia alone is seldom described. We evaluated an opioid-free protocol combining pectoral nerves (PECS I–II) and parasternal blocks with intravenous sedation.

**Methods:**

Between January 2020 and December 2024, 627 women (mean age 35 y) underwent bilateral primary augmentation under ultrasound-guided fascial-plane blocks plus sedation. VAS pain scores were recorded at predefined intervals up to 72 h. Descriptive statistics, cubic modelling of pain trajectories and Kruskal–Wallis tests compared implant planes; cumulative pain was expressed as the 0–72 h area under the curve (AUC).

**Results:**

Mean VAS was < 2 in all groups by 72 h. Pain peaked at 16 h, highest with dual-plane placement (mean 7.0) and lower with submuscular and pre-pectoral placements (both ≈ 5.5; *p *< 0.001). Inter-group differences persisted to 48 h but disappeared by 72 h (*p *= 0.115). AUC confirmed the greatest pain burden for dual-plane (292 VAS·h) versus submuscular (238) and pre-pectoral (200). Implant volume showed only a weak overall correlation with pain (ρ = 0.20) and none within individual planes. No patient required rescue opioids; most resumed normal activities within four days, and no major complications occurred.

**Conclusions:**

Ultrasound-guided PECS I–II and parasternal blocks with sedation provide safe, effective, opioid-free analgesia for aesthetic breast augmentation. Early pain is determined mainly by the implant plane, not by implant size, and should be considered in surgical planning and postoperative care.

**Level of Evidence III:**

This journal requires that authors assign a level of evidence to each article. For a full description of these Evidence-Based Medicine ratings, please refer to the Table of Contents or the online Instructions to Authors www.springer.com/00266.

## Introduction

Breast augmentation is one of the most frequently performed procedures in aesthetic plastic surgery. In 2023 alone, over 1.8 million cosmetic breast augmentations were performed worldwide. , with a significant proportion involving subpectoral implant placement [1]. When implants are inserted in a subpectoral plane, manipulation of the pectoralis major muscle can result in moderate to severe postoperative pain, which may delay recovery and increase reliance on systemic analgesics [2]. Although breast augmentation is one of the most performed aesthetic procedures, postoperative pain management remains highly variable, with no universally accepted protocol. Despite enhanced recovery after surgery (ERAS) recommendations for multimodal analgesia and opioid-sparing strategies in breast surgery, their implementation is still inconsistent in clinical practice [3]. Recent prospective data in outpatient aesthetic breast procedures under sedation confirm low opioid use but also highlight the absence of a standardized analgesic regimen [4, 5].

Traditionally, postoperative pain has been managed with paracetamol, nonsteroidal anti-inflammatory drugs (NSAIDs) and opioid medications. However, opioid-related adverse events (ORAEs) such as nausea, vomiting, pruritus, sedation, and urinary retention can compromise patient comfort and prolong hospital stay [6]. Moreover, in outpatient settings, excessive reliance on opioids may hinder early discharge and functional recovery [7]. Consequently, there is growing interest in regional anesthesia techniques that can reduce or eliminate the need for systemic opioids.

Pectoral nerve blocks (PECS I–II blocks) were introduced in 2011 as a regional anesthesia technique for breast surgery [8]. PECS I involves the injection of local anesthetic between the pectoralis major and minor muscles, targeting the lateral and medial pectoral nerves. PECS II builds upon this by adding a second injection between the pectoralis minor and serratus anterior muscles to anesthetize the intercostal nerve and the long thoracic nerve [9]. PECs blocks belong to a broader category of ultrasound-guided interfascial plane blocks developed for thoracic wall surgery, including the paravertebral, intercostal and serratus anterior plane blocks [10]. These blocks have shown promising results in breast surgery, particularly, reducing postoperative pain and opioid consumption. However, evidence supporting their efficacy in aesthetic breast augmentation – especially when combined with mild sedation and performed without general anesthesia – remains limited [11].

We retrospectively evaluated ultrasound-guided PECS I–II and parasternal blocks under sedation for pain control, analgesic use and recovery time. This approach may support the development of enhanced recovery protocols in aesthetic surgery by enabling early discharge and improving overall patient experience.

## Materials and Methods

This retrospective study included 627 female patients aged between 18 and 55 years who underwent primary bilateral breast augmentation between January 2020 and December 2024 at a private outpatient surgical clinic. All procedures were performed by the same senior plastic surgeon to ensure homogeneity in surgical technique and patients were managed perioperatively by the same anesthesiologist. All data were anonymized prior to analysis, and the study was conducted under the Declaration of Helsinki. Given the retrospective design of the study, the use of fully anonymized routinely collected data and the absence of any experimental intervention or deviation from standard clinical care, formal ethical committee review was not required. Written informed consent for the surgical and anesthetic procedures, including permission for the anonymized use of clinical data for research purposed was obtained from all patients.

The inclusion criteria were primary aesthetic breast augmentation. Exclusion criteria included previous breast or thoracic surgery, chronic pain-related disorders (such as rheumatologic, neurologic or psychiatric conditions), chronic use of opioids, antidepressants, or anticonvulsants, known allergy to local anesthetics, history of bleeding diathesis or ongoing anticoagulant therapy, major cardiopulmonary disorders, hepatic or renal impairment, chest wall deformities, pregnancy or breastfeeding and refusal to undergo regional anesthesia [12, 13].

Implant placement was performed in subpectoral, submuscular or prepectoral planes, depending on individual anatomical characteristics and clinical indications [14, 15]. For each patient, the implant volume was also recorded and considered as a variable in the postoperative outcome analysis [16].

**Fig. 1:**
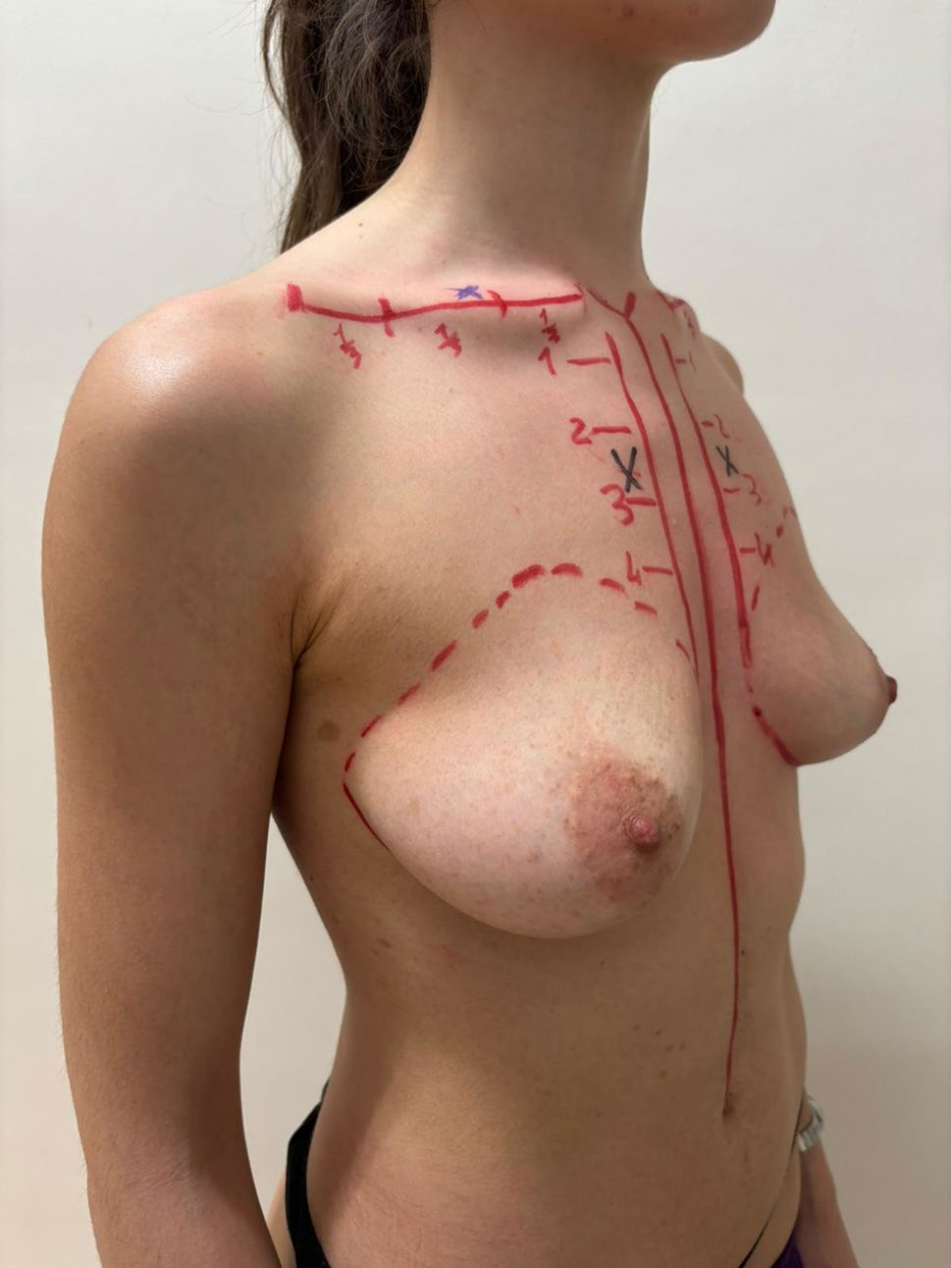
Surface landmarks and skin entry points for ultrasound-guided blocks. Blue mark: common skin entry point for PECS I–II. Black mark: parasternal block entry point (second intercostal space)

**Fig. 2:**
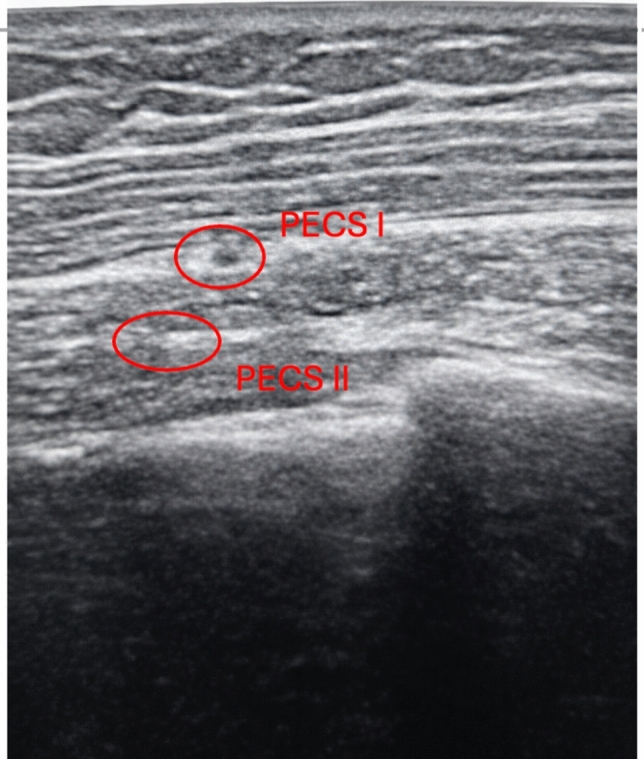
Representative ultrasound image illustrating the target interfascial planes for PECS I and PECS II injections

**Fig. 3:**
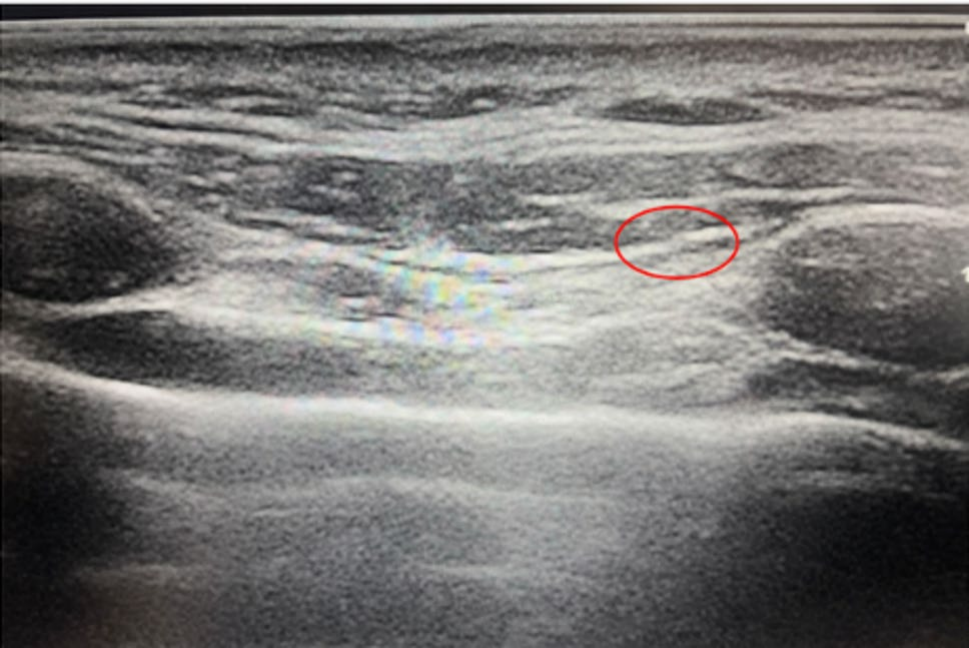
Representative ultrasound image illustrating the target parasternal injection plane at the second intercostal space

All patients received low-dose intravenous midazolam (1–2 mg) as premedication prior to the regional block procedure, to minimize anxiety and discomfort during needle insertion and injection [17]. PECS and parasternal blocks were performed bilaterally under aseptic conditions using 12 MHz linear ultrasound probe. For both PECS I and PECS II, the same skin entry point was used, with the probe positioned in the sagittal plane above the medial third of the clavicle. The parasternal block was performed separately at the second intercostal space. (Fig. 1) [18] Representative ultrasound images illustrating the target interfascial planes for PECS I–II and the parasternal block are provided in Figs. 2 and 3. A 22-gauge, 50-mm sharp-tip needle was advanced into the interfascial plane, and correct placement was confirmed by hydrodissection with 2 ml of sterile 0.9% saline. A total of 70 ml of local anesthetic solution (35 ml per side) was administered: 10 ml for PECS I (between pectoralis major and minor), 20 ml for PECS II (between pectoralis minor and serratus anterior) and 5 ml for parasternal block (between the pectoralis major and external intercostal muscle, at the third and fourth costal cartilages). The anesthetic solution consisted of levobupivacaine 2 mg/kg and ropivacaine 3 mg/kg, plus 4 mg dexamethasone as an adjuvant to prolong analgesia. Epinephrine was not added to the local anesthetic solution to avoid adrenergic effects during intravenous sedation and because operative times were short [19, 20].

All patients underwent intravenous sedation, to minimize discomfort from surgical traction and facilitate implant insertion. Sedation was induced and maintained with propofol (administered either as TIVA or TCI, associated with midazolam or dexmedetomide). No intraoperative opioids were administered.

Adverse events were recorded and divided as sedation-related (e.g. nausea, vomiting, pruritus, excessive sedation, respiratory or hemodynamic events) or block-related (e.g. ecchymosis, hematoma, pneumothorax, hemothorax, local anesthetic systemic toxicity).

Postoperative pain was assessed using the Visual Analogue Scale (VAS), where 0 represents no pain and 10 represents the worst imaginable pain. VAS scores were recorded at 1, 2, 4, 8, 16, 36, 48, and 72 hours postoperatively. Postoperative analgesia included paracetamol 1000 mg every 8 hours for the first 72 hours and thiocolchicoside 4 mg intramuscularly once daily for 3 consecutive days, administered to all patients. NSAIDs (ibuprofen 600 mg every 8 hours, up to 1800 mg/day) were administered only when VAS ≥ 3. No opioid medications were required.

Recovery outcomes (time to discharge, resumption of daily activities, return to work, analgesic use and adverse events) were recorded descriptively. VAS scores were summarized for each implant plane (mean, median and IQR) at every scheduled time-point; between-group differences were assessed with the Kruskal–Wallis test, adopting *p *< 0.05 as the threshold for significance. Pain trajectories were illustrated by fitting a third-degree (cubic) polynomial to the mean VAS profile of each plane. Cumulative pain burden was expressed as the 0–72 h area under the curve (AUC), calculated with the trapezoidal rule for each plane. The relationship between implant volume and early pain (VAS at 16 h) was explored with Spearman’s rank correlation for the entire cohort and within each plane. No missing data were recorded at any follow-up point.

## Results

A total of 627 patients were included in the analysis. The mean patient age was 35 years (range: 18–55), and the mean BMI was 22.3 kg/m^2^ (range: 19.4–25.2). Implant placement was performed in a dual plane in 67.9% of cases (426/627), submuscular in 17.9% (112/627), and prepectoral in 14.2% (89/627). The mean implant volume was 389 ± 68 cc (95% CI 383–396) for the dual plane group, 286 ± 23 cc (95% CI 282–290) for the submuscular group and 412 ± 87 cc (95% CI 394–430) for the prepectoral group. Corresponding median values (IQR) were 390 cc (335-450), 285 cc (265-300) and 410 cc (340-495), respectively (Table 1).

**Table 1:** Baseline implant characteristics by surgical plane

Implant plane	n	% of total	Mean (cc)	SD	95 % CI low	95 % CI high	Median (cc)	IQR low	IQR high
Dual-plane	426	67,9	389	68	383	396	390	335	450
Submuscular	112	17,9	286	23	282	290	285	265	300
Pre-pectoral	89	14,2	412	87	394	430	410	340	495

**Fig. 4:**
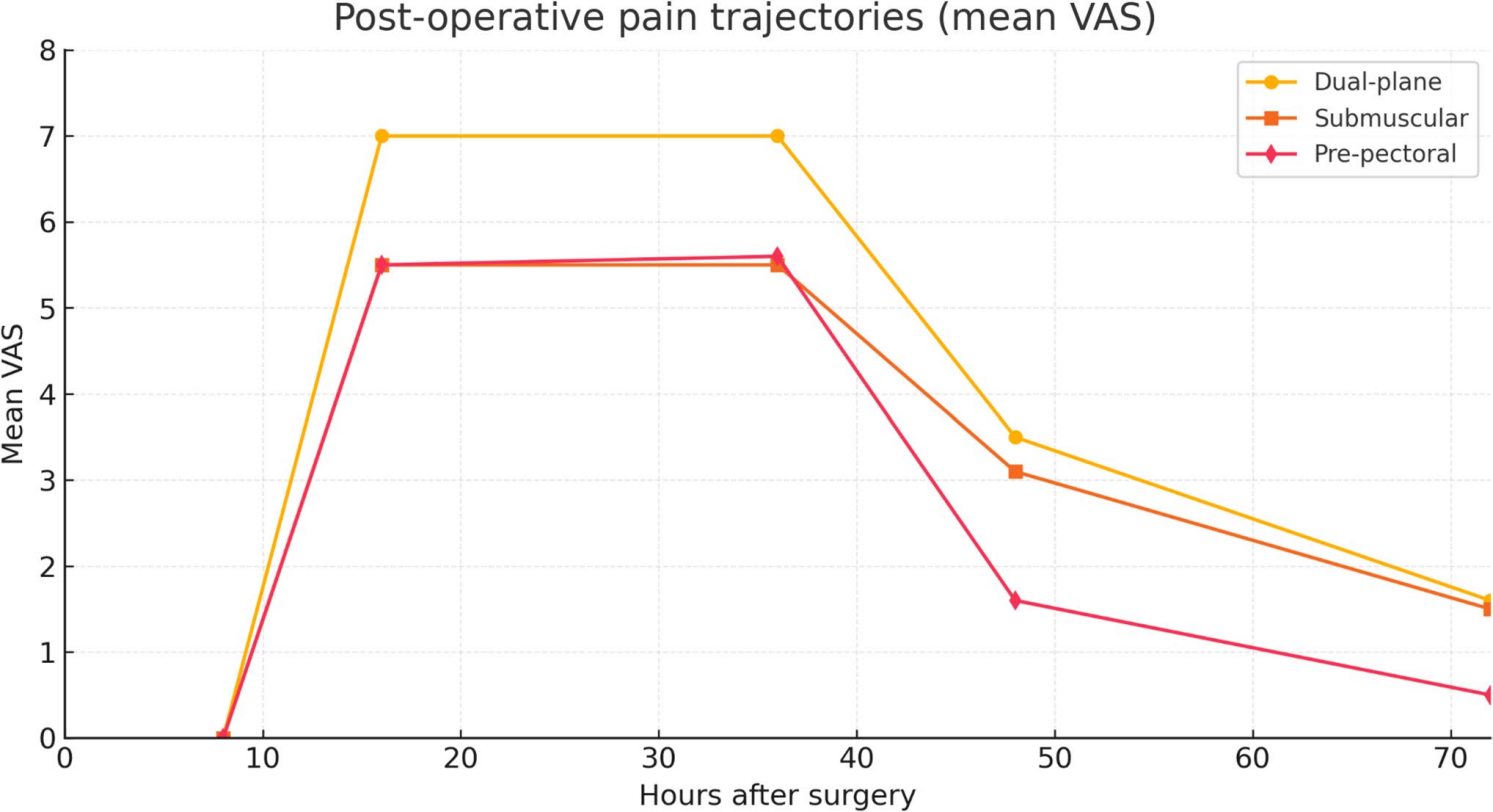
Post-operative pain trajectories (mean VAS)

Post-operative pain, measured with the Visual Analogue Scale (VAS), showed a similar temporal pattern across the three implantation planes. During the first 8 hours all patients reported a VAS of 0. Pain peaked at 16 hours, highest in the dual-plane group (mean 7.0; range 6–8), followed by the submuscular (mean 5.5; range 5–6) and pre-pectoral groups (mean 5.5; range 5–6). Scores remained essentially unchanged at 36 hours (dual-plane 7.0; submuscular 5.5; pre-pectoral 5.6). By 48 hours, pain declined markedly to 3.5 (range 2–5), 3.1 (2–4) and 1.6 (0–3), respectively, and further subsided at 72 hours to 1.6 (1–2), 1.5 (1–2) and 0.5 (0–1), data are summarized in Table 2 and Fig. 4.

**Table 2:** Mean VAS scores at predefined time-points (0-72h)

Time (h)	Dual-plane	Submuscular	Pre-pectoral
8	0	0	0
16	7	5,5	5,5
36	7	5,5	5,6
48	3,5	3,1	1,6
72	1,6	1,5	0,5

The VAS medians followed the same downward trajectory observed for the means (Table 2).

Statistical comparison with the Kruskal–Wallis test demonstrated significant differences in pain scores among the three surgical planes at 16, 36 and 48 hours (all *p *< 0.001). No significant difference persisted at 72 hours (*p *= 0.115), indicating comparable pain resolution across groups beyond the second postoperative day. These findings confirm that implant placement in the dual-plane position is associated with the highest early postoperative discomfort, whereas inter-group differences disappear by 72 hours.

To quantify the overall pain exposure, the area under the curve (AUC) of the mean VAS–time profile (0–72 h) was calculated for each surgical plane using the trapezoidal rule. The AUC was 292 VAS·h for dual-plane, 238 VAS·h for submuscular and 200 VAS·h for pre-pectoral placement, confirming that dual-plane augmentation carries the greatest cumulative pain burden over the first 72 hours after surgery.

Pain evolution over time was modelled for each implant group with a cubic polynomial, yielding excellent goodness-of-fit (adjusted R^2 ^= 0.96 for dual-plane, 0.98 for submuscular and 0.83 for pre-pectoral), thereby providing a concise and reliable description of the postoperative pain course.

Implant volume showed a weak positive correlation with pain at 16 hours when the entire cohort was analyzed (Spearman’s ρ = 0.20, *p *< 0.001). No significant association was detected within any individual surgical plane (dual-plane ρ = − 0.05, *p *= 0.30; submuscular ρ = 0.12, *p *= 0.22; pre-pectoral ρ = − 0.14, *p *= 0.19), indicating that implant size per se does not account for early postoperative pain, which is primarily influenced by the plane of placement.

All patients were discharged 6 hours after surgery. Return to daily activities occurred after a mean of 4 days, while return to work was achieved within 15 days.

No major adverse events were observed. With respect to sedation-related events, no clinically relevant respiratory depression, airway intervention, aspiration or hemodynamic instability (hypotension or bradycardia requiring treatment) was recorded. Minor sedation-related side effects occurred in a minority of cases, including mild nausea (3.3%) and transient dizziness (2.2%). Regarding regional anesthesia, no serious block-related complications were recorded, including pneumothorax, hemothorax or local anesthetic systemic toxicity (LAST). Minor local events included puncture-site ecchymosis (7.3%) and subcutaneous hematoma (3.4%), both managed conservatively. In terms of block performance, incomplete sensory coverage was recorded in 9.6% of cases, whereas an unintended sensory spread, including involvement of the long thoracic nerve territory, was observed in 2.6% of patients.

## Discussion

This study assessed 627 women who underwent bilateral aesthetic breast augmentation under ultrasound-guided PECS I, PECS II and parasternal blocks plus intravenous sedation, avoiding general anesthesia. Pain trajectories diverged by implant plane: VAS peaked at 16 h with means of 7.0 for dual-plane and 5.5 for submuscular and pre-pectoral placements, remained different through 48 h (*p *< 0.001) and fell below 2 in all groups by 72 h. Corresponding 0–72 h pain AUCs were 292, 238 and 200 VAS·h. No opioids were required. Paracetamol and thiocolchicoside facilitated discharge within 6 h and functional recovery in 15 days. No major adverse events occurred; follow-up was complete for every enrolled patient.

Previous randomized data demonstrated that PECS I block alone may be insufficient for effective analgesia after breast surgery [21]. In contrast, our protocol combined PECS I, PECS II and parasternal blocks, targeting both medial and lateral chest wall innervation likely contributing to its higher efficacy. PECS I targets the medial and lateral pectoral nerves between the major and minor muscles, while PECS II extends the blockade to lateral cutaneous branches of intercostal nerves and the long thoracic nerve. When combined with parasternal infiltration, this approach ensures extensive analgesia of the anterior thoracic wall—adequate for all implant placement planes, including submuscular, dual plane, and prepectoral techniques.

While PECS blocks have been widely used in oncologic breast surgery, their application in aesthetic augmentation without general anesthesia remains underreported [22, 23]. A systematic review confirmed the growing use of PECS and serratus anterior blocks in breast surgery, but emphasized the predominance of oncologic indications [24]. A randomized trial comparing PECS I+II to placebo in dual plane augmentation under general anesthesia demonstrated significant reductions in postoperative VAS and opioid consumption yet did not evaluate the blocks as a standalone strategy [11]. To date, no published studies have assessed PECS blocks combined with parasternal infiltration and intravenous sedation—without general anesthesia—in a large cohort of patients undergoing aesthetic bilateral breast augmentation.

The absence of general anesthesia and intraoperative opioids in our study further underscores the safety and feasibility of this protocol in an ambulatory setting. None of the typical opioid-related adverse effects (e.g., sedation, nausea, urinary retention) were observed, while pain remained well controlled with non-opioid agents and fascial plane blocks. In addition, the choice of regional anesthesia over systemic opioids may contribute to reducing the incidence of persistent postoperative pain, which has been reported in up to 20% of patients after cosmetic breast surgery—especially with submuscular implants [25] Early and effective pain control likely plays a role in preventing chronic pain syndromes by reducing central sensitization.

Alternative techniques such as thoracic paravertebral block and epidural analgesia, although effective, are associated with higher technical complexity and risk of severe complications like dural puncture or pneumothorax [26, 27]. Intercostal nerve blocks, on the other hand, require multiple injections and are not always sufficient in this context [28]. Fascial plane blocks offer a safer and more reproducible alternative, especially when guided by ultrasound. While serratus anterior plane blocks have been described for more lateral coverage, they are rarely necessary in aesthetic cases without axillary dissection.

The link between implant position and postoperative pain is increasingly recognized. Elevating the pectoralis major, as required in sub- or dual-plane augmentation, produces more nociceptive input than a purely pre-pectoral approach, translating into higher early VAS scores and a slower decline in pain—a pattern reproduced in our dual-plane cohort (mean VAS 7.0 at 16 h vs. ≈ 5.5 in the other planes), together with the largest 0–72 h AUC (292 VAS·h) [29].

By contrast, the effect of implant size on acute pain is less clear. Biomechanically, larger devices can increase soft-tissue stretch and muscular tension, potentially intensifying discomfort [30]. In our series, implant volume showed only a weak overall correlation with VAS at 16 h (ρ = 0.20) and no correlation within individual planes, indicating that plane of placement—rather than size—is the primary driver of early pain.

These findings underscore the need to factor implant plane into pre-operative planning when optimizing postoperative comfort; implant volume appears to play a secondary role in acute analgesic requirements and recovery trajectories after aesthetic breast augmentation.

Only 37 of the 627 patients (5.9%) in our series reported mild nausea or dizziness postoperatively, and all were managed conservatively. Interestingly, previous studies had not shown a strong correlation between PECS blocks and reduced incidence of postoperative nausea and vomiting; however, in our protocol, the absence of general anesthesia and opioid use likely contributed to the low rate of these symptoms.

In addition to regional anesthesia, all patients received postoperative paracetamol and thiocolchicoside as part of a standardized protocol. Previous regimens combining systemic agents such as NSAIDs and acetaminophen have demonstrated opioid-sparing effects, yet lacked the anatomical specificity provided by regional blocks [31]. Although evidence remains limited in aesthetic surgery, the rationale for administering a muscle relaxant lies in its ability to counteract postoperative pectoralis major muscle spasm. In postoperative settings, muscle relaxants have been shown to reduce myofascial pain and muscle spam, thereby improving early functional recovery in orthopedic and visceral surgery. This strategy may support improved early comfort and further minimized the pain after surgery. Future studies should explore its isolated role with multimodal pain management protocols [32, 33].

Taken together, these results support the adoption of an opioid-free, block-based anesthesia protocol for aesthetic breast augmentation. The combination of targeted fascial plane blocks, mild intravenous sedation, and adjunctive agents such as paracetamol and thiocolchicoside enabled effective pain control, rapid discharge, and early return to daily life—avoiding general anesthesia or opioids. These advantages are relevant in ambulatory settings or in patients with contraindications to systemic opioids or airway management.

This retrospective single-center study lacks a general anesthesia control group. Pain data were limited to the first 72 h; longer follow-up would be required to explore chronic pain. Because every patient received the same intravenous sedation regimen, the independent contribution of sedation to analgesia could not be analyzed. Future prospective, randomized trials with an appropriate control group are needed to clarify long-term pain, recovery and patient-satisfaction outcomes.

## Conclusion

Ultrasound-guided PECS I–II blocks combined with parasternal infiltration and intravenous sedation constitute a safe, opioid-free alternative to general anesthesia for bilateral aesthetic breast augmentation. The protocol yielded predictable analgesia—pain peaked at 16 h, fell below VAS 2 by 72 h in all patients—without rescue opioids, major adverse events or delayed recovery.

Early discomfort was driven chiefly by the implant plane: dual-plane placement produced higher peak VAS scores and the largest 0–72 h pain AUC, whereas implant volume showed only a weak, non-independent relation to pain. Thiocolchicoside may have contributed to the smooth postoperative course by limiting muscular spasm, but its specific effect remains to be clarified.

Because acute pain is a recognized predictor of chronic postoperative pain, early regional analgesia could provide longer-term benefits. Prospective, controlled studies should compare this block-and-sedation strategy with conventional general anesthesia, isolate the role of adjunctive muscle relaxants and evaluate its impact on long-term pain, function and patient satisfaction.
